# Early administration of remdesivir to COVID-19 patients associates with higher recovery rate and lower need for ICU admission: A retrospective cohort study

**DOI:** 10.1371/journal.pone.0258643

**Published:** 2021-10-26

**Authors:** Hawra Ali Hussain Alsayed, Fatemeh Saheb Sharif-Askari, Narjes Saheb Sharif-Askari, Ali Al Sayed Hussain, Qutayba Hamid, Rabih Halwani

**Affiliations:** 1 Pharmacy Department, Dubai Health Authority, Dubai, United Arab Emirates; 2 Sharjah Institute of Medical Research, University of Sharjah, Sharjah, United Arab Emirates; 3 Department of Clinical Sciences, College of Medicine, University of Sharjah, Sharjah, United Arab Emirates; 4 Meakins-Christie Laboratories, Research Institute of the McGill University Health Center, Montreal, Quebec, Canada; 5 Prince Abdullah Ben Khaled Celiac Disease Chair, Department of Pediatrics, Faculty of Medicine, King Saud University, Riyadh, Saudi Arabia; Ohio State University Wexner Medical Center Department of Surgery, UNITED STATES

## Abstract

**Objectives:**

Remdesivir is one of the most widely recommended and used medications for COVID-19 treatment. However, different outcomes have been reported for hospitalized patients with COVID-19 treated with remdesivir. Specifically, the effect of the timing of remdesivir initiation (from patient’s symptom onset) on clinical outcomes in COVID-19 patients has not been investigated.

**Methods:**

This is a retrospective cohort study of patients hospitalized with COVID-19 and treated with or without remdisivir. The primary outcome was patient’s recovery rate, defined as clinical improvement and patient’s discharge by day 14 of symptom onset. The secondary outcome was the need for intensive care unit (ICU) admission, mechanical ventilation, and mortality within 28 days of patient’s symptom onset.

**Results:**

Out of 323 hospitalized adults with COVID-19, 107 (33.1%) received no remdesivir during their hospital stay, 107 (33.1%) received remdesivir early within 7 days of the symptom onset, and 109 (33.7%) received it at 8 days or later of symptom onset. At day 14 following symptom onset, higher proportion of patients recovered in the early remdesivir compared to the late remdesivir cohort, or patients who did not receive remdesivir (adjusted odds ratio, aOR, 2.65; 95% confidence interval [CI], 1.31 to 5.35). Moreover, early administration of remdesivir was associated with lower admission to intensive care unit (adjusted hazard ratio [aHR], 0.31; 95% CI, 0.15 to 0.64), less need for mechanical ventilation (aHR, 0.22; 95% CI, 0.10 to 0.51), and lower mortality at 28 days (aHR, 0.15; 95% CI, 0.04 to 0.53), as compared to the late remdesivir cohort or patients who did not receive remdesivir.

**Conclusion:**

Early administration of remdesivir within 7 days of symptom onset is associated with less need for mechanical ventilation and lower 28-days mortality.

## Introduction

COVID-19, a respiratory illness caused by severe acute respiratory syndrome coronavirus 2 (SARS- CoV-2), was first identified in December 2019 in Wuhan city, China [1]. So far, several supportive therapies and anti-viral treatment have been investigated for the treatment of COVID-19. Dexamethasone has been shown to decrease mortality compared to placebo (25.7% in the placebo versus 22.9% in the dexamethasone cohort; P = 0.001), with the largest benefit seen among patients receiving mechanical ventilation support [2]. Biologics, such as anti-interleukin (IL)-6 receptor monoclonal antibody, tocilizumab was also used in combination to steroids, especially for the treatment of moderate to severe COVID-19 cases [3, 4]. Among the anti-viral drugs, protease inhibitors, Lopinavir-ritonavir have failed to demonstrate efficacy in several COVID-19 clinical trials [4–6]. However, remdesivir, an inhibitor of viral RNA-dependent-RNA polymerase, was shown to have invitro inhibitory activity against SARS-CoV-1 [7], Middle East respiratory syndrome [8], and SARS-CoV-2 [9]. It is the only WHO-approved antiviral treatment that has shown to reduce recovery time in severe COVID-19 [4, 10–12], and hence it is now the most widely used anti-SARS-CoV-2 treatment.

Several randomized trials have been published on the use of remdesivir in patients with COVID-19. In a randomized clinical trial by Beigel et al. [10], remdesivir has been reported to shorten the time to recovery in patients who received it within 10 days of their symptom onset. Furthermore, remdesivir was associated with mortality benefit (HR, 0.28; 95% CI, 0.12 to 0.66) when given early in the course of illness and/or in patients with less severe disease who require low-flow oxygen, but not in those receiving high-flow oxygen (HR, 0.82; 95% CI, 0.40 to 1.69) or mechanical ventilation (HR, 0.98; 95% CI, 0.70 to 1.36). Similarly, in the Adaptive COVID-19 Treatment Trial (ACTT-1) [10], remdesivir showed a reduction in time to recovery and mortality rate in patients who required low-flow oxygen (HR, 0.30; 95% CI, 0.11 to 0.81) but not those needed mechanical ventilation (HR, 1.13; 95% CI, 0.57 to 2.23). However, in SOLIDARITY therapeutic trials [13], while it reduced the time to recovery when given early in patients with less severe disease, it had lower mortality benefit (HR, 0.86; 95% CI, 0.67 to 1.11). Overall, remdesivir might not significantly affect mortality rate of patients hospitalized with COVID-19, but that it does reduce time to recovery when given early in the course of disease. There is also a possible mortality benefit in such patients, which could be understood better with further research.

The effect of time from symptom onset to start of treatment on disease progression is well established. However, the influence of time from symptom onset to start of remdesivir treatment on its efficacy to reduce disease progression and mortality rate has not been investigated.

Thus, in this retrospective cohort study we evaluated the effect of timing of remdesivir treatment from patient’s symptom onset on patient’s clinical outcomes. We studied the proportion of: 1) recovered patients on day 14, 2) patients needed mechanical ventilation, and 3) mortality rate of hospitalized patients with confirmed COVID-19 infection.

## Materials and methods

### Ethics statement

Ethical approval was obtained from the Dubai Scientific Research Ethics Committee (DSREC), Dubai Health Authority at Rashid Hospital (DSREC-12/2020_02). In this study all patients’ records were analyzed in a fully anonymized and de-identified manner and no researcher had access to patients’ personal information, thus no consent was obtained.

### Study cohort

In this retrospective cohort study, adults with laboratory-confirmed SARS-CoV-2 infection by reverse transcription-quantitative polymerase chain reaction (RT-qPCR), who were hospitalized during September 2020, and January 2021, were included. Patients were divided into the following groups: (1) no remdesivir cohort, patients who received no remdesivir during the hospital stay owing to elevated alanine transaminase (ALT) or aspartate transaminase (AST) values, or an estimated glomerular filtration rate (eGFR) less than 30 mL/min/1.73 m^3^; (2) early remdesivir cohort, patients who received remdesivir within 7 days from the symptom onset, and (3) late remdesivir cohort, patients who received remdesivir at 8 days or later from the symptom onset, mostly due to their delayed hospital admission. Furthermore, neither physicians nor patients influenced the allocation of patients to these treatment groups except within the context of the national treatment protocols, and therefore the study was less susceptible to selection bias or confounding by indication.

The United Arab Emirates (UAE) national guidelines for clinical management and treatment of COVID-19 [14], recommends remdesivir administration to patients with COVID-19 pneumonia with SpO2 ≤94% on room air, or on supplemental oxygen, but not on mechanical ventilation or ECMO (Extracorporeal membrane oxygenation). It also limits its administration to patients with ALT and AST values less than 5 times the upper limit of normal and an eGFR greater than 30 mL/min/1.73 m^3^.

Remdesivir was administered intravenously as a 200-mg loading dose on day 1, followed by a 100-mg maintenance dose administered daily for up to a median of 6 additional days or until hospital discharge or death. We have evaluated the effect of timing of remdesivir initiation from patient’s clinical outcomes.

### Outcomes

The first outcome was the proportion of recovered patients on day 14, defined as clinical improvement and patient discharge by day 14 of symptom onset. The time from onset of illness to discharge was adopted from previous clinical reports of patients with COVID-19 (an estimate average recovery time of 14 days after symptom onset) [15–17]. Other outcomes were the need for intensive care unit (ICU) admission, mechanical ventilation, as well as mortality within 28 days.

### Statistical analysis

Chi-square or Fisher exact tests for categorical variables, and Student *t* test or Mann-Whitney *U* test for continuous variables, depending on the skewness of data were used to compare baseline clinical characteristics between early and late remdesivir cohorts. For estimation of the odds ratio of recovered patients at 14 days, logistic regression analysis was used adjusted for patients age, gender, baseline body mass index (BMI), SpO2 on admission, diabetes mellitus, use of tocilizumab, and COVID-19 severity status. For ICU admission, need of mechanical ventilation, or mortality at 28 days, Cox proportional hazard models were developed, adjusted for patients age, gender, baseline BMI, SpO2 on admission, diabetes mellitus, use of tocilizumab, and COVID-19 severity status. Kaplan–Meier survival curves were then constructed to show cumulative survival over the 28-day period. Statistical analysis was performed using SPSS (version 26.0), R software (version 3.6.1) and PRISM (version 8). All tests were two-tailed and a P value of less than 0.05 was considered statistically significant. A file consisting of all patient’s parameters used in the analysis is provided in S1 File.

## Results

Of the 323 recruited patients, 107 received no remdesivir, 107 received remdesivir early within 7 days of symptoms onset, and 109 patients received remdesivir late at 8 days or later following their symptom’s onset. All patients were followed through day 28. The mean (standard deviation [SD]) age of the patients was 52 (12) years and 74% were males. Most patients had either one (34.7%) or two or more (37.7%) of the prespecified coexisting conditions at enrollment, most commonly cardiovascular disease including hypertension (41.8%), type 2 diabetes mellitus (40.6%), and obesity (38.4%). There was no difference in COVID-19 severity related laboratory markers such as CRP, D-Dimer, and ferritin serum levels between no remdesivir and early or late remdesivir groups on admission. However, patients who received no remdesivir had higher AST value and relatively lower eGFR on admission as compared to early or late remdesivir groups (Table 1). There were no differences in ALT (*P* = 0.976) and AST (*P* = 0.349) values, or eGFR rate (*P* = 0.550) between early or late remdesivir groups at admission.

**Table 1:** Baseline clinical characteristics of the study population.

	No remdesivir (n = 107)	Early remdesivir (n = 107)	Late remdesivir (n = 109)	*P-value*
Age, years, median (IQR), y	54 (45–65)	51 (42–56)	51 (43–59)	0.004
Sex				
Men	83 (78)	77 (72)	81 (74)	0.639
Women	24 (22)	30 (28)	28 (26)	
SpO2	93 (85–96)	94 (91–96)	94 (92–96)	0.578
Time from start of symptom onset to first dose of remdesivir, days	-	5 (3–6)	10 (9–12)	<0.0001
Duration of remdesivir, days	-	6 (5–7)	5 (5–7)	0.073
**Underlying comorbidities**				
Diabetes	54 (50.5)	38 (36)	39 (36)	0.038
Cerebrovascular disease	54 (50.5)	36 (34)	45 (41)	0.044
Obesity	43 (40)	34 (32)	47 (43)	0.093
Smoker	5 (2)	8 (11)	12 (14)	0.115
**Baseline laboratory findings (normal range)**				
White cell count (3.9–11.10 x 10^9^ per L)	7 (5.05–9.1)	6.5 (5.1–8.9)	6.3 (5.2–8.4)	0.401
C-reactive protein (1.0–3.0 mg/L)	105.2 (40.8–168.3)	80.1 (38.8–157.4)	80.4 (28.5–137.4)	0.147
IL-6	63.7 (23.3–135)	30.5 (7.6–69.6)	27.2 (7.5–62.1)	0.548
D-Dimer (0–0.5 μ/mL)	1.01 (0.6–1.79)	0.74 (0.50–1.31)	0.68 (0.47–1.11)	0.252
Ferritin (10–204 ng/mL)	682.4 (328.4–1473)	730.3 (338–1567)	644.7 (334–1231.3)	0.517
Alanine transaminase (0–55 U/L)	36 (22.5–65)	47 (31–73.5)	45 (31–75.5)	0.027
Aspartate transaminase (5–34 U/L)	45 (29.5–68)	27 (20–37)	27 (22–46)	<0.0001
Estimated glomerular filtration rate, (90–120 mL/min/1.73 m^2^)	88.1 (68.4–104.4)	102 (93–112)	104 (94–113)	<0.0001

Data are n (%) or median (IQR). Abbreviation: COVID-19, coronavirus disease 2019.

Moreover, there was no difference between early and late remdesivir groups in COVID-19 severity status, defined as COVID-19 pneumonia requiring high-flow oxygen therapy or non-invasive ventilation (16% and 22% of patients in early and late remdesivir groups, respectively; P = 0.292) [S1 Table]. Additionally, there was no difference in the use of other antiviral therapies such as favipiravir, interferon-beta-1b, triple combination of interferon beta-1b, lopinavir–ritonavir, and ribavirin, or lopinavir–ritonavir between these 3 treatment groups (S1 Table). However, for the use of supportive medications, tocilizumab was used more in the no remdesivir group compared to early and late remdesivir groups (51%, 34%, and 38% of patients in the no remdesivir, early and late remdesivir groups, respectively; P = 0.024). All the patients received systemic corticosteroids such as dexamethasone or methylprednisolone (S1 Table).

**Fig 1:**
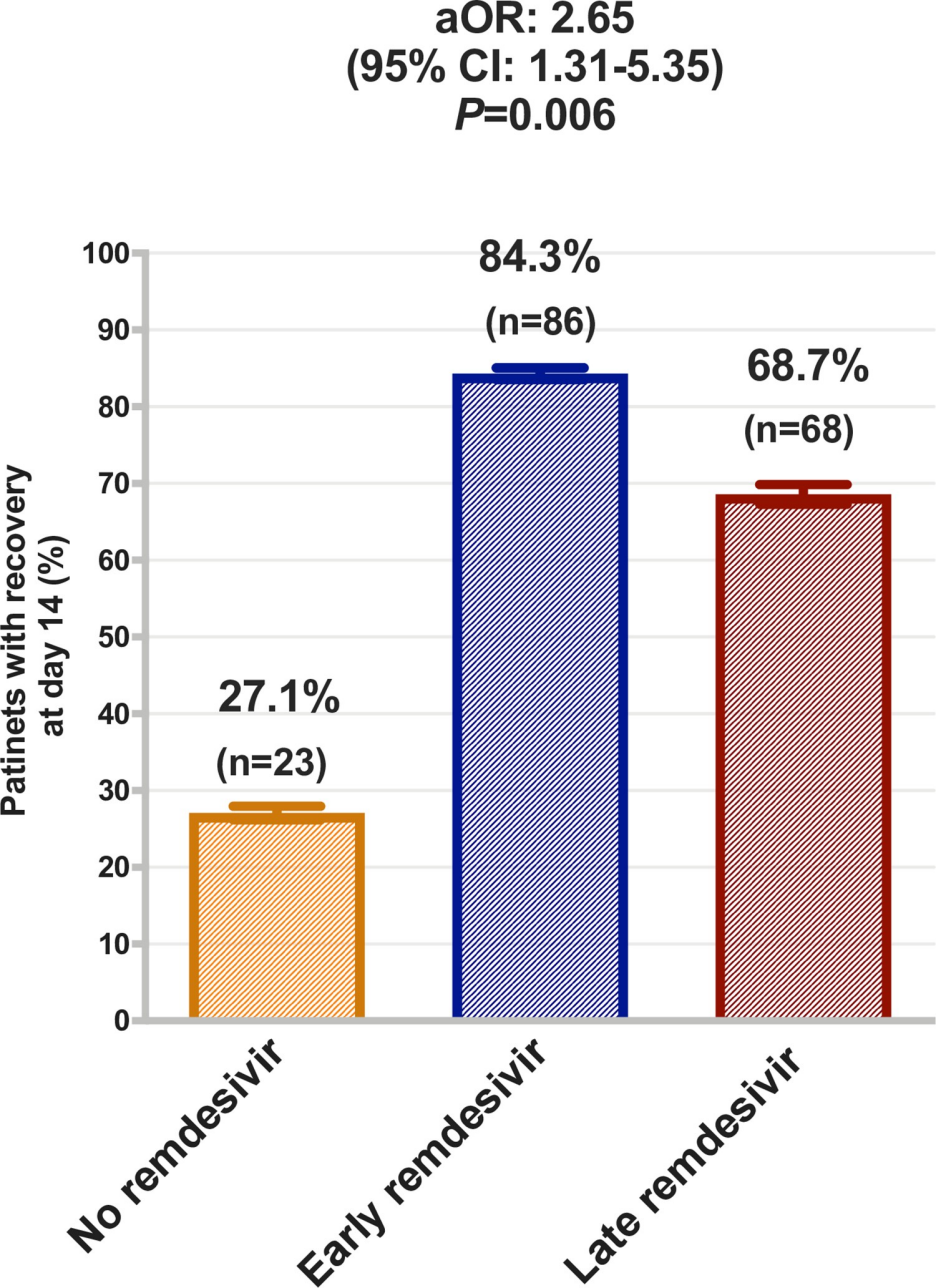
Proportion (95% confidence interval) of patients recovered at day 14 in the no remdesivir, early remdesivir, and late remdesivir-cohort. Recovery was defined as improvement and discharged home by day 14. Logistic regression model was adjusted for patients age, gender, baseline body mass index, SpO2 on admission, diabetes mellitus, use of tocilizumab, and COVID-19 severity status.

Furthermore, the median time to recovery from COVID-19 was 13 days after symptom onset and it ranged from 8–26 days. Interestingly, the proportion of recovered patients on day 14 after the symptom onset were higher in early remdesivir 84.3% (n = 86) compared to the late remdesivir cohort 68.7% (n = 68), and in the no remdesivir group 27.1% (n = 23) (Adjusted odds ratio [aOR], 2.65; 95% CI, 1.31 to 5.35; *P* = 0.006) [Fig 1].

**Fig 2:**
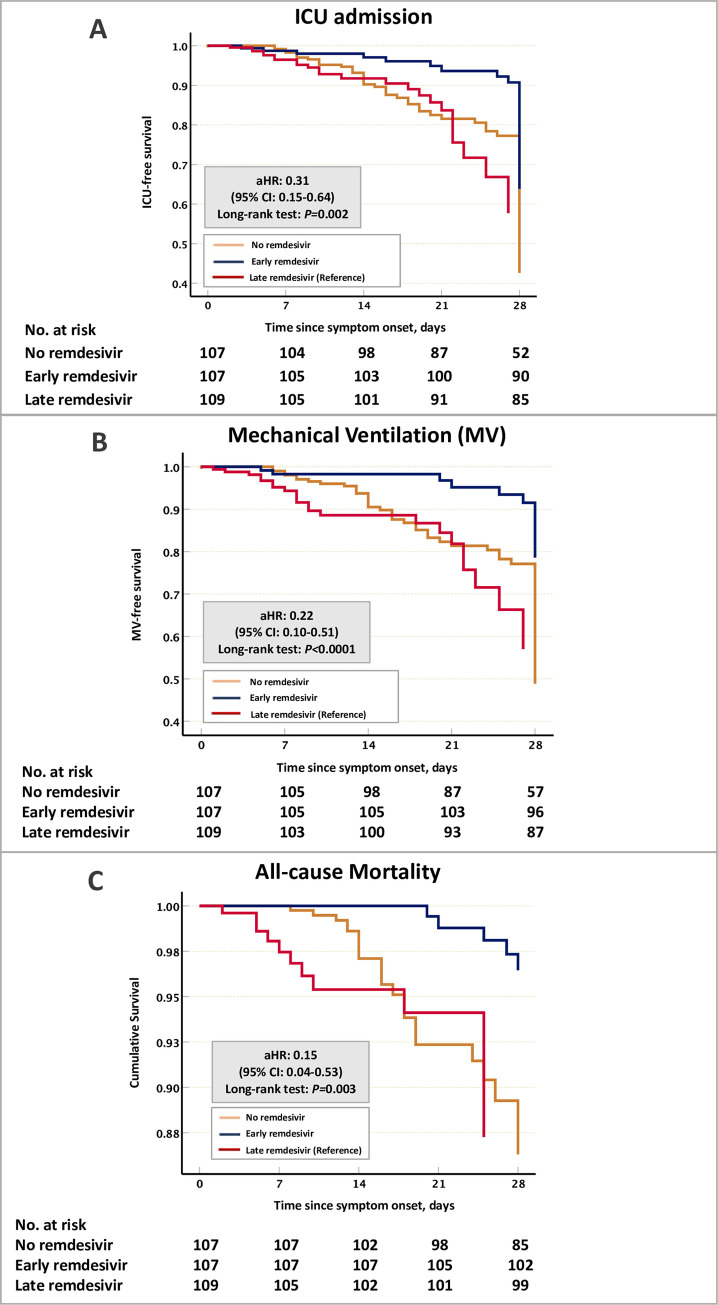
Kaplan–Meier survival analysis of the ICU admission, need for mechanical ventilation, and 28-day mortality in the no remdesivir, early remdesivir, and late remdesivir-cohort. Cox proportional model analysis adjusted with patients age, gender, baseline body mass index, SpO2 on admission, diabetes mellitus, use of tocilizumab, and COVID-19 severity status. Abbreviation: COVID-19, coronavirus disease 2019; ICU, intensive care unit.

In addition, the early remdesivir cohort were associated with lower ICU admission compared to late remdesivir cohort or no remdesivir group (15.9% (n = 17) in early remdesivir, 22% (n = 24) in late remdesivir, and 51.4% (n = 55) in no remdesivir group; adjusted hazard ratio [aHR], 0.31; 95% CI, 0.15 to 0.64; *P* = 0.002) [Fig 2A]. The early remdesivir cohort were also associated with less need for mechanical ventilation compared to late remdesivir cohort or no remdesivir group (11.6% (n = 11) in early remdesivir, 21.8% (n = 22) in late remdesivir, and 46.7% (n = 50) in no remdesivir group; aHR, 0.22; 95% CI, 0.10 to 0.51; *P*<0.0001) [Fig 2B]. Moreover, the early remdesivir cohort had lower all-cause mortality compared to late remdesivir cohort or no remdesivir group (4.7% (n = 5) in early remdesivir, 9.2% (n = 10) in late remdesivir, and 20.6% (n = 22) in no remdesivir group; aHR, 0.15; 95% CI, 0.04 to 0.53; *P* = 0.003) [Fig 2C]. These results indicate that early remdesivir administration was associated with a higher recovery rate, lower ICU admission, need for mechanical ventilation, and 28-days mortality rate.

## Discussion

In this retrospective cohort study, our findings suggested that early treatment with remdesivir within 7 days of symptom onset has improved clinical status of patients with COVID-19 pneumonia, and prevented their progression to more severe respiratory disease. This was apparent by the lower proportion of patients admitted to ICU, as well as needing higher levels of respiratory support such as mechanical ventilation.

In a clinical trial that was evaluating the effect of the timing of remdesivir initiation on clinical outcome [11], patients were receiving either remdesivir within or after 10 days of symptom onset. Although not statistically significant, remdesivir within 10 days from symptom onset was associated with a shortened time to clinical improvement and lower 28-day mortality. In addition, in a clinical trial by Beigel et al. [10] the recovery and clinical improvement were statistically significant when patients were randomized to remdesivir vs placebo within 9 days of symptom onset. In our study, early administration of remdesivir within 7 days of symptom onset significantly increased the proportion of patients recovered at 14 days, reduced the need for ICU admission and mechanical ventilation, and lowered 28-day mortality of COVID-19 hospitalized patients. Cumulatively, these further highlight the benefit of early remdesivir administration to hospitalized COVID-19 patients.

Moreover, building on the preclinical efficacy of remdesivir in animal models of SARS-COV-2 infection, initiating remdesivir treatment early during infection significantly reduced viral loads in lung tissue [6], increasing its efficacy against the acute infections [18].

Beigel et al. [10], suggested that the benefit of remdesivir treatment was most apparent in patients with mild disease, those receiving low-flow oxygen at baseline. Here, in our study there was no difference in COVID-19 related severity laboratory markers, such as serum levels of CRP, D-dimers, ferritin, and IL-6, as well as oxygen saturation level at baseline among patients who received remdesivir early or late during hospital stay. However, further studies are needed to associate patient disease status during early administration of remdesivir with clinical outcomes.

Furthermore, Spinner et al. [19] have shown that patients who received remdesivir for 5 days had higher odds of clinical improvement than those receiving placebo (odds ratio, 1.65; 95% CI, 1.09 to 2.48; P = 0.02). In the same study, no additional benefit was seen with the 10-day course (P = 0.18). In our study, however, all patients were on a median of 6 days on the remdesivir therapy with no significant difference in the number of days between early and late remdesivir groups.

Similar to any observational study, this investigation has few limitations. Our study was not a randomized controlled trial, although by development of multivariate regression models, the effect of observed confounders was adjusted. However, there might be a number of unobservable confounders that could only be controlled with a randomized controlled trial. Therefore we cannot completely rule out the effect of unobserved or residual confounding factors or treatment selection bias. Moreover, this study was limited to a single hospital in UAE which may also limit the generalization of the results.

Despite the various combination therapies used, the mortality rate from COVID-19 is still globally high ranging from 3 to 24% [20, 21]. Remdesivir is usually given in combination with several immunotherapy agents such baricitinib (Janus kinase inhibitor) and interferon beta-1a [18, 22]. Although a variety of therapeutic approaches including novel antivirals, several immunotherapy agents, and combination treatments are needed, our findings highlight the importance of treatment timing and urges for more research to investigate that for other medications and treatment strategies.

## Supporting information

S1 File Study raw data. (SAV)

S1 Table Supportive and anti-viral medications in no remdesivir, early remdesivir, and late remdesivir-cohort. (PDF)

## References

[1] Helmy YA, Fawzy M, Elaswad A, Sobieh A, Kenney SP, Shehata AA. The COVID-19 pandemic: a comprehensive review of taxonomy, genetics, epidemiology, diagnosis, treatment, and control. Journal of clinical medicine. 2020;9(4):1225. 10.3390/jcm9041225 PMC7230578 32344679

[2] Dexamethasone in Hospitalized Patients with Covid-19. New England Journal of Medicine. 2020;384(8):693–704. doi: 10.1056/NEJMoa2021436 32678530 PMC7383595

[3] Rosas IO, Bräu N, Waters M, Go RC, Hunter BD, Bhagani S, et al. Tocilizumab in Hospitalized Patients with Severe Covid-19 Pneumonia. New England Journal of Medicine. 2021. doi: 10.1056/NEJMoa2028700 33631066 PMC7953459

[4] Pan H, Peto R, Henao-Restrepo AM, Preziosi MP, Sathiyamoorthy V, Abdool Karim Q, et al. Repurposed Antiviral Drugs for Covid-19—Interim WHO Solidarity Trial Results. N Engl J Med. 2021;384(6):497–511. Epub 2020/12/03. doi: 10.1056/NEJMoa2023184 ; PubMed Central PMCID: PMC7727327. 33264556 PMC7727327

[5] Cao B, Wang Y, Wen D, Liu W, Wang J, Fan G, et al. A Trial of Lopinavir-Ritonavir in Adults Hospitalized with Severe Covid-19. N Engl J Med. 2020;382(19):1787–99. Epub 2020/03/19. doi: 10.1056/NEJMoa2001282 ; PubMed Central PMCID: PMC7121492. 32187464 PMC7121492

[6] Williamson BN, Feldmann F, Schwarz B, Meade-White K, Porter DP, Schulz J, et al. Clinical benefit of remdesivir in rhesus macaques infected with SARS-CoV-2. Nature. 2020;585(7824):273–6. doi: 10.1038/s41586-020-2423-5 32516797 PMC7486271

[7] Sheahan TP, Sims AC, Graham RL, Menachery VD, Gralinski LE, Case JB, et al. Broad-spectrum antiviral GS-5734 inhibits both epidemic and zoonotic coronaviruses. Science translational medicine. 2017;9(396). doi: 10.1126/scitranslmed.aal3653 28659436 PMC5567817

[8] Sheahan TP, Sims AC, Leist SR, Schäfer A, Won J, Brown AJ, et al. Comparative therapeutic efficacy of remdesivir and combination lopinavir, ritonavir, and interferon beta against MERS-CoV. Nature communications. 2020;11(1):1–14. doi: 10.1038/s41467-019-13993-7 31924756 PMC6954302

[9] Wang M, Cao R, Zhang L, Yang X, Liu J, Xu M, et al. Remdesivir and chloroquine effectively inhibit the recently emerged novel coronavirus (2019-nCoV) in vitro. Cell Res. 2020;30(3):269–71. doi: 10.1038/s41422-020-0282-0 32020029 PMC7054408

[10] Beigel JH, Tomashek KM, Dodd LE, Mehta AK, Zingman BS, Kalil AC, et al. Remdesivir for the Treatment of Covid-19—Final Report. New England Journal of Medicine. 2020;383(19):1813–26. doi: 10.1056/NEJMoa2007764 32445440 PMC7262788

[11] Wang Y, Zhang D, Du G, Du R, Zhao J, Jin Y, et al. Remdesivir in adults with severe COVID-19: a randomised, double-blind, placebo-controlled, multicentre trial. The Lancet. 2020;395(10236):1569–78. doi: 10.1016/S0140-6736(20)31022-9 32423584 PMC7190303

[12] Rochwerg B, Agarwal A, Zeng L, Leo YS, Appiah JA, Agoritsas T, et al. Remdesivir for severe covid-19: a clinical practice guideline. Bmj. 2020;370:m2924. Epub 2020/08/01. doi: 10.1136/bmj.m2924 . 32732352

[13] Repurposed Antiviral Drugs for Covid-19—Interim WHO Solidarity Trial Results. New England Journal of Medicine. 2020;384(6):497–511. doi: 10.1056/NEJMoa2023184 33264556 PMC7727327

[14] National Guidelines for Clinical Management and Treatment of COVID-19, https://www.dha.gov.ae/en/HealthRegulation/Documents/NationalGuidelinesforClinicalManagementandTreatmentofCOVID-19.pdf, 13 April, 2020.

[15] Bhapkar HR, Mahalle PN, Dey N, Santosh KC. Revisited COVID-19 Mortality and Recovery Rates: Are we Missing Recovery Time Period? Journal of Medical Systems. 2020;44(12):202. doi: 10.1007/s10916-020-01668-6 33099706 PMC7585674

[16] Olender SA, Perez KK, Go AS, Balani B, Price-Haywood EG, Shah NS, et al. Remdesivir for Severe Coronavirus Disease 2019 (COVID-19) Versus a Cohort Receiving Standard of Care. Clinical Infectious Diseases. 2020. doi: 10.1093/cid/ciaa1041 32706859 PMC7454434

[17] Interim Guidance on Ending Isolation and Precautions for Adults with COVID-19, https://www.cdc.gov/coronavirus/2019-ncov/hcp/duration-isolation.html, Access date: 14/7/2021.

[18] Sheahan TP, Sims AC, Zhou S, Graham RL, Pruijssers AJ, Agostini ML, et al. An orally bioavailable broad-spectrum antiviral inhibits SARS-CoV-2 in human airway epithelial cell cultures and multiple coronaviruses in mice. Science translational medicine. 2020;12(541). doi: 10.1126/scitranslmed.abb5883 32253226 PMC7164393

[19] Spinner CD, Gottlieb RL, Criner GJ, López JRA, Cattelan AM, Viladomiu AS, et al. Effect of remdesivir vs standard care on clinical status at 11 days in patients with moderate COVID-19: a randomized clinical trial. Jama. 2020;324(11):1048–57. doi: 10.1001/jama.2020.16349 32821939 PMC7442954

[20] Verity R, Okell LC, Dorigatti I, Winskill P, Whittaker C, Imai N, et al. Estimates of the severity of coronavirus disease 2019: a model-based analysis. The Lancet infectious diseases. 2020;20(6):669–77. doi: 10.1016/S1473-3099(20)30243-7 32240634 PMC7158570

[21] Zhou F, Yu T, Du R, Fan G, Liu Y, Liu Z, et al. Clinical course and risk factors for mortality of adult inpatients with COVID-19 in Wuhan, China: a retrospective cohort study. The lancet. 2020;395(10229):1054–62. doi: 10.1016/S0140-6736(20)30566-3 32171076 PMC7270627

[22] Richardson P, Griffin I, Tucker C, Smith D, Oechsle O, Phelan A, et al. Baricitinib as potential treatment for 2019-nCoV acute respiratory disease. The Lancet. 2020;395(10223):e30–e1. doi: 10.1016/S0140-6736(20)30304-4 32032529 PMC7137985

