# Supplementary material for: Early administration of remdesivir to COVID-19 patients associates with higher recovery rate and lower need for ICU admission: A retrospective cohort study
Source: PLoS One. 2021 Oct 26;16(10):e0258643. doi: 10.1371/journal.pone.0258643 (PMC8547637; doi:10.1371/journal.pone.0258643)
Supplement: S1 Table — (PDF) [file pone.0258643.s002.pdf]

S2 Table. Supportive and anti-viral medications in the early remdesivir and late remdesivir-cohort

	**No remdesivir (n=107)**	**Early remdesivir (n=107)**	**Late remdesivir (n=109)**	***P-value***
COVID-19 severity status[@]	51 (48)	17 (16)	24 (22)	0.001
Supportive medications[#]				
Tocilizumab	55 (51)	36 (34)	42 (38)	0.024
Antiviral medications*				
Favipiravir	20 (19)	8 (8)	14 (13)	0.051
Interferon beta-1b	19 (18)	10 (10)	10 (11)	0.088
Interferon beta-1b and favipiravir	19 (18)	7 (7)	16 (15)	0.042
Interferon beta-1b, lopinavir–ritonavir, and ribavirin	13 (12)	5 (5)	10 (9)	0.147
Lopinavir–ritonavir	12 (11)	4 (4)	8 (7)	0.114

Data are n (%). [@] COVID-19 severity status is defined as a COVID-19 pneumonia requiring high-flow oxygen therapy or non-invasive ventilation. [#]All the patients received systemic corticosteroids including dexamethasone 6 mg daily, or methylprednisolone with 2 mg/kg/day starting dose and max 60 mg/day for pulse dosing. *Antiviral therapies before the initiation of remdesivir. Abbreviation: COVID-19, coronavirus disease 2019.
